# Supplementary material for: PCMT1 knockdown attenuates malignant properties by globally regulating transcriptome profiles in triple-negative breast cancer cells
Source: PeerJ. 2023 Nov 6;11:e16006. doi: 10.7717/peerj.16006 (PMC10634331; doi:10.7717/peerj.16006)
Supplement: Figure S1 [file peerj-11-16006-s001.docx]

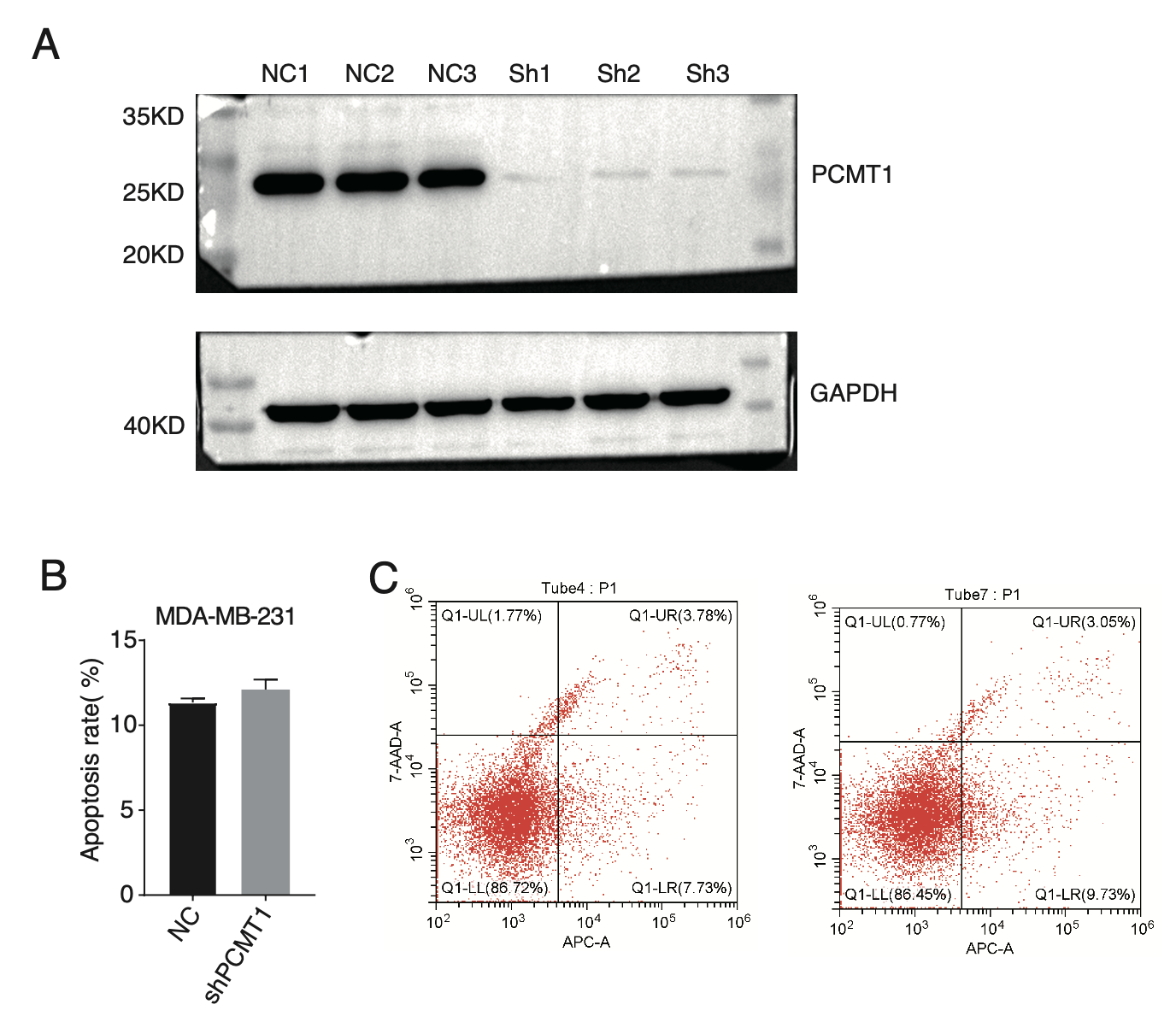


**Figure S1. PCMT1 knockdown influences MDA-MB-231 cell apoptosis.**

1. Raw and uncutted western blot result of PCMT1 knockdown in MDA-MB-231 cells.
2. Bar plot showed the apoptosis results of MDA-MB-231 cells after PCMT1 knockdown. N = 3.
3. Flow-type presentation of the apoptosis result between shPCMT1 and NC samples.


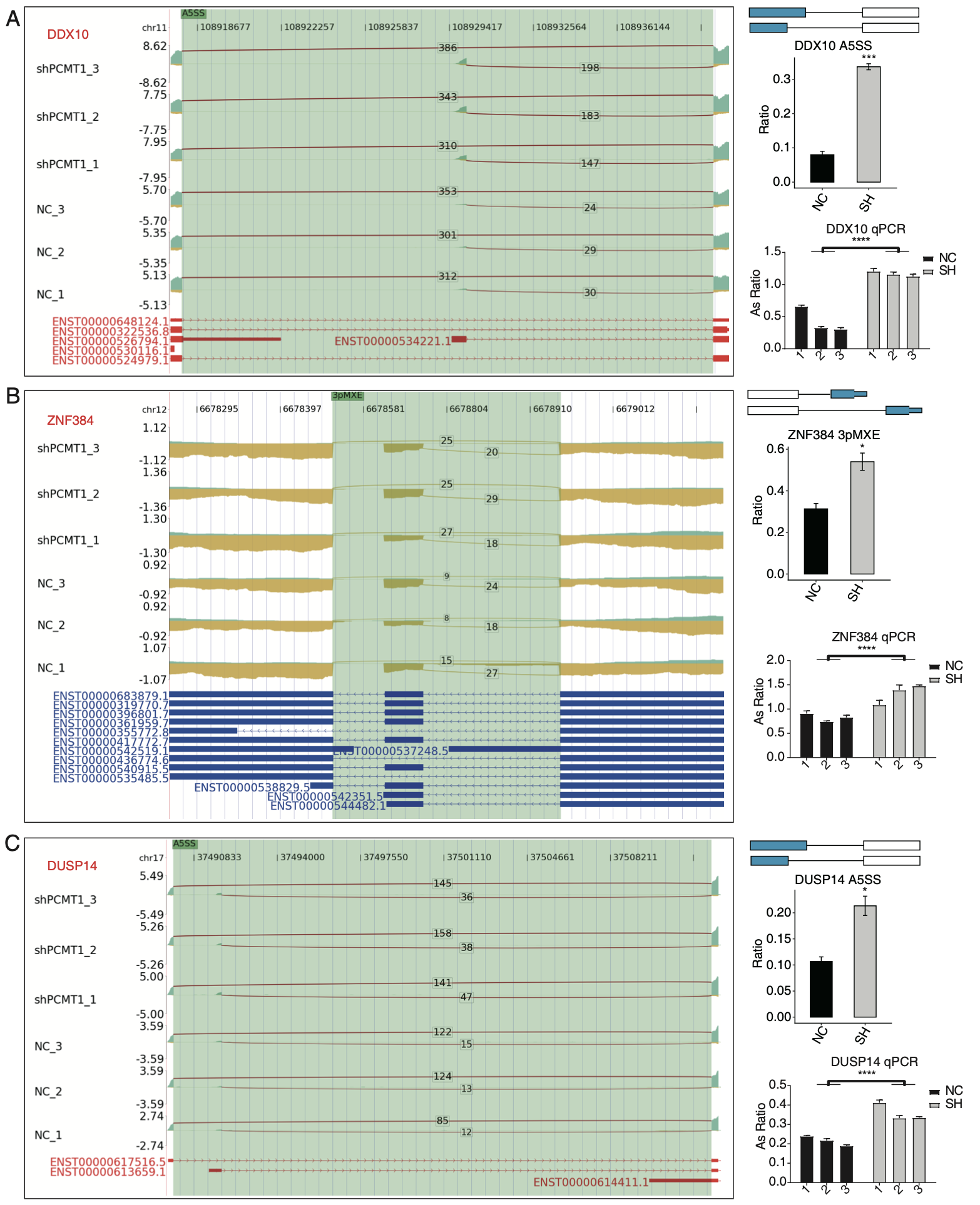


**Figure S2. PCMT1 regulates the AS of *DDX10*, *ZNF384*, and *DUSP14* in MDA-MB-231 cells.**

1. PCMT1 regulates the AS of *DDX10*. Left panel: IGV-sashimi plot shows the RASEs and binding sites across mRNAs. Reads distribution of RASE is shown above and the transcripts of each gene are shown below. Right panel: The schematic diagram depicts the structure of ASEs. RNA-seq and RT-qPCR validation of ASEs was shown in the right middle and bottom panel, respectively. Error bars represent mean ± SEM. N = 3. *** *p*-value < 0.001, **** *p*-value < 0.0001; Student’s *t*-test.
2. The same as (A) but for *ZNF384*. Error bars represent mean ± SEM. N = 3. * *p*-value < 0.05, **** *p*-value < 0.0001; Student’s *t*-test.
3. The same as (A) but for *DUSP14*. Error bars represent mean ± SEM. N = 3. * *p*-value < 0.05, **** *p*-value < 0.0001; Student’s *t*-test.

**
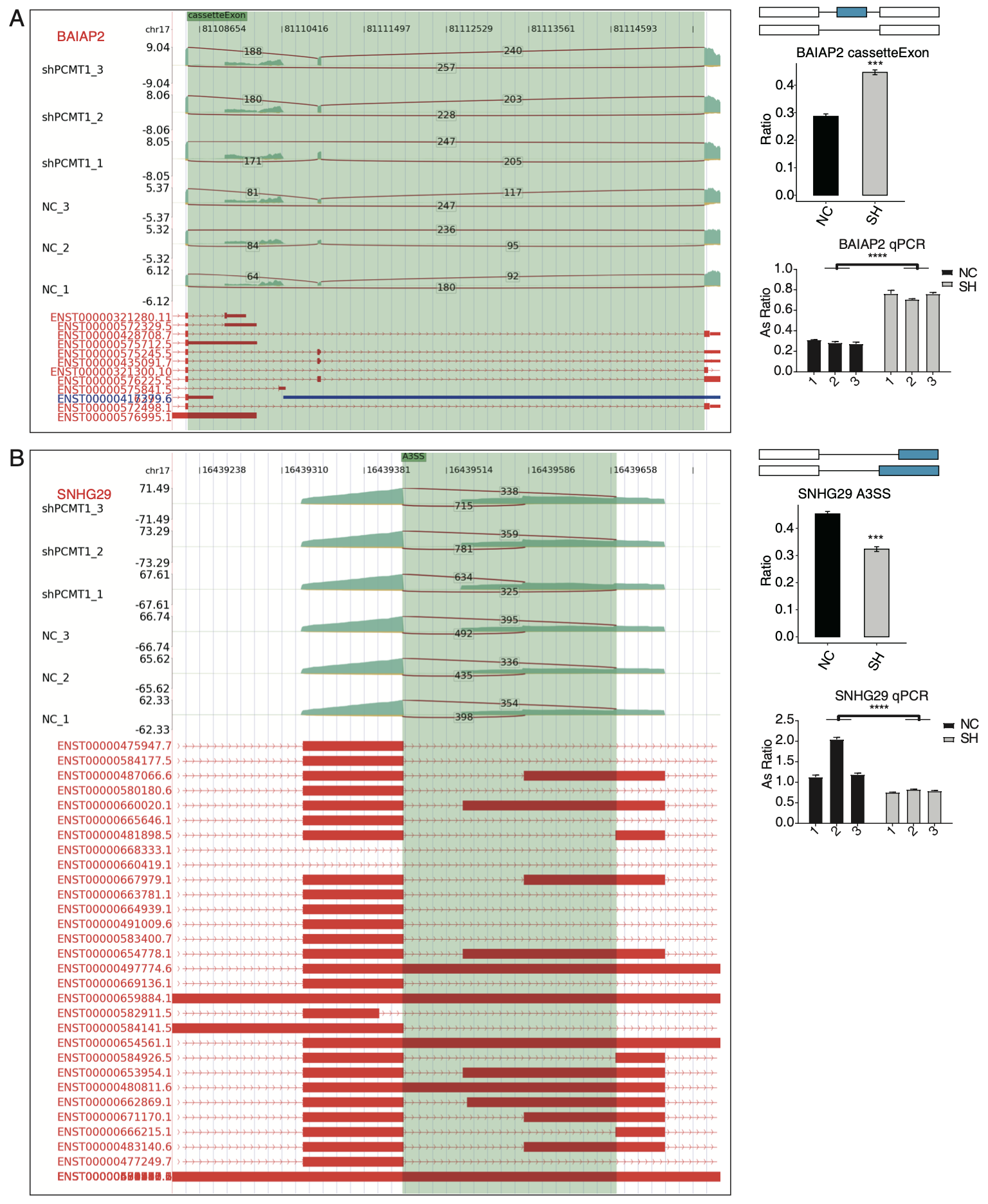
**

**Figure S3. PCMT1 regulates the AS of *BAIAP2* and *SNHG29* in MDA-MB-231 cells.**

1. PCMT1 regulates the AS of *BAIAP2*. Left panel: IGV-sashimi plot shows the RASEs and binding sites across mRNAs. Reads distribution of RASE is shown above and the transcripts of each gene are shown below. Right panel: The schematic diagram depicts the structure of ASEs. RNA-seq and RT-qPCR validation of ASEs was shown in the right middle and bottom panel, respectively. Error bars represent mean ± SEM. N = 3. *** *p*-value < 0.001, **** *p*-value < 0.0001; Student’s *t*-test.
2. The same as (A) but for *SNHG29*. Error bars represent mean ± SEM. N = 3. *** *p*-value < 0.001, **** *p*-value < 0.0001; Student’s *t*-test.


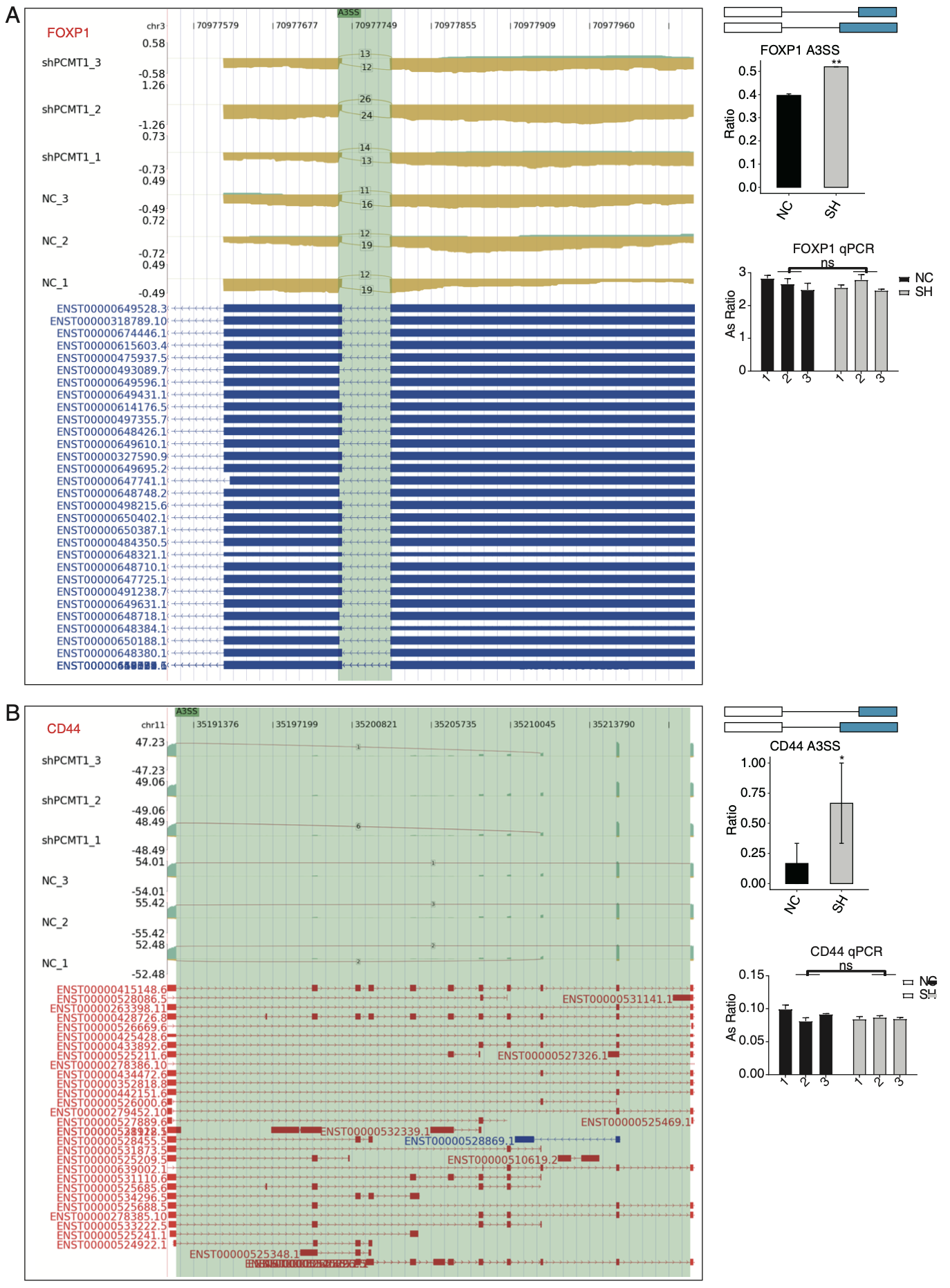


**Figure S4. PCMT1 regulates the AS of *FOXP1* and *CD44* in MDA-MB-231 cells.**

1. PCMT1 regulates the AS of *FOXP1*. Left panel: IGV-sashimi plot shows the RASEs and binding sites across mRNAs. Reads distribution of RASE is shown above and the transcripts of each gene are shown below. Right panel: The schematic diagram depicts the structure of ASEs. RNA-seq and RT-qPCR validation of ASEs was shown in the right middle and bottom panel, respectively. Error bars represent mean ± SEM. N = 3. ** *p*-value < 0.01; Student’s *t*-test.
2. The same as (A) but for *CD44*. Error bars represent mean ± SEM. N = 3. * *p*-value < 0.05; Student’s *t*-test.
