# Supplementary material for: PCMT1 knockdown attenuates malignant properties by globally regulating transcriptome profiles in triple-negative breast cancer cells
Source: PeerJ. 2023 Nov 6;11:e16006. doi: 10.7717/peerj.16006 (PMC10634331; doi:10.7717/peerj.16006)
Supplement: Table S1 [file peerj-11-16006-s002.docx]

Internal control：

hum-GAPDH-F： GGTCGGAGTCAACGGATTTG

hum-GAPDH-R： GGAAGATGGTGATGGGATTTC

| ZC3H12A-F | CCTCCTCTACGCCAATGA |
| --- | --- |
| ZC3H12A-R | GGCAGCAGGATAAGGAATAG |
| TACSTD2-F | GGCAATGTCTGTCCTCAA |
| TACSTD2-R | GCCTATGTAGTAGCCTCATT |
| S100A4-F | CCAACCACATCAGAGGAG |
| S100A4-R | AGATGAAGCTGCTTTCCA |
| EDIL3-F | ACACAGCTTGAAGGTTTG |
| EDIL3-R | GAAGACTTGGGTTACTAATG |
| SPDEF-F | CACGAACTGGTAGACGAG |
| SPDEF-R | CGCCATGAACTACGACAA |
| FAM20C-F | ACAGATGCCAATCACCTAC |
| FAM20C-R | CAGGAAGGAGAAAGGGTTT |
| PEG10-F | TGGGAGAAAGTTCAAAGGT |
| PEG10-R | GGTTCAGTGGTAGGAGGA |
| OLFML2A-F | GCTTAGTGAGGACAAGACC |
| OLFML2A-R | TTGACTCTACTGCCTGTTG |
| ADM-F | TGGAATAAGTGGGCTCTGA |
| ADM-R | AATAAGGGTCTGGGCAGG |
| RBMS2-F | AGGCTGGAGGGTAATACAT |
| RBMS2-R | GGTGGCATTGGCATAGAT |
| ESRP2-F | TTGCCATCTGTTACTCCTTA |
| ESRP2-R | TCCCTGTCCCTCTTGTAG |
| NQO1-F | CTAAAGCAAGTCAGGGAAG |
| NQO1-R | AGGTACAGGATGAGGAGAA |
| SPNS2-F | GTAAGCAGGTGGAATACTCA |
| SPNS2-R | AAGGAAGAGGAGGCAACA |
| CCL2-F | CCAGTCACCTGCTGTTAT |
| CCL2-R | CAGCTTCTTTGGGACACT |
| FSTL3-F | ACGGAGGGTCTAGTCTGA |
| FSTL3-R | GGAAGCTGGAGTTCTTGG |
| EEF1A2-F | GGTACTGGGAGAAGCTCT |
| EEF1A2-R | GCTGAAGGAGAAGATTGAC |
| DDX10-M-F | ATAAAATAAGTGATACCAAG |
| DDX10-AS-F | GATGGGAAAGTGATACCAAG |
| DDX10-M/AS-R | CAAAGGCTCTAAAGTGTCCC |
| AHNAK-M-F | TGTGGTGTGTTCAGAACCACTT |
| AHNAK-AS-F | ATCATCCCCGCTCAGAACCACTT |
| AHNAK-M/AS-R | CCTGAAGCTGCACCGCAAGG |
| SNHG29-M/AS-F | CAGAGCCTGGAGTCTGCGAAGG |
| SNHG29-M-R | GTTTCAGCGACCAGTTCTCT |
| SNHG29-AS-R | GGTACGCAATCCAGTTCTCT |
| DUSP14-M-F | CGCCGCGCCGATTTGATTTGTAT |
| DUSP14-AS-F | TTGACTGCAGATTTGATTTGTAT |
| DUSP14-M/AS-R | CCAGAAAGTCCTAAGTGAGC |
| BAIAP2-M-F | TCCATGAGCAGGAATCCCTTT |
| BAIAP2-AS-F | ACTAGAGTTAGAATCCCTTT |
| BAIAP2-M/AS-R | CAGATGATGACAGAACAGGCTACA |
| ITGA6-AS-F | ACTATGGAAGTGTGGATTCT |
| ITGA6-M-F | TCTGTAATTGTGTGGATTCT |
| ITGA6-M/AS-R | CCCGCTATGAGTAGCTTTCA |
| FOXP1-AS-F | TGCGCAATATCTGACGAAAT |
| FOXP1-M-F | GCAATATCTGCTGACGAAAT |
| FOXP1-M/AS-R | CCCTCTGTCATCACAACCACC |
| NPM1-M/AS-F | AAAAGGACCTAGTTCTGTAG |
| NPM1-AS-R | TTCAATGCGCTTTTTCTATA |
| NPM1-M-R | GAGAACCACCTTTTTCTATA |
| CD44-M/AS-F | GCAACCCTACTGATGATGAC |
| CD44-AS-R | CTTCTTCCACTGGTAGCAGG |
| CD44-M-R | ATGTGAGTGTCTGGTAGCAGG |
| ZNF384-AS-F | AAACCCGGACCAGCAGTCAT |
| ZNF384-M-F | AACAGGAGACTCCAGCAGTCAT |
| ZNF384-M/AS-R | CAGCCAAGCGTCCGTTACCC |
